# Editorial: Current advances in Fusarium wilt

**DOI:** 10.3389/fpls.2025.1683977

**Published:** 2025-09-23

**Authors:** Mahyar Mirmajlessi, Inmaculada Larena, María Del Mar Guerrero, María del Carmen Rodríguez-Molina

**Affiliations:** ^1^ Department of Plants and Crops, Faculty of Bioscience Engineering, Ghent University, Ghent, Belgium; ^2^ Department of Plant Protection, Instituto de Investigación y Tecnología Agraria y Alimentaria-CSIC, Madrid, Spain; ^3^ Instituto Murciano de Investigacion y Desarrollo Agrario y Medioambiental, Mayor La Alberca Murcia, Spain; ^4^ Centro de Agricultura Ecologica y de Montana (CAEM), Centro de Investigaciones Cientificas y Tecnologicas de Extremadura (CICYTEX), Plasencia, Spain

**Keywords:** *Fusarium* wilt, harnessing antagonistic fungi and soil amendments, bioactive plant compounds, microbial interactions and community dynamics, integrating host resistance and genomic approaches

*Fusarium* wilt, caused by a diverse group of *Fusarium* spp., remains one of the most formidable soilborne threats to agricultural productivity worldwide. The persistence of its propagules in soil, its broad host range, and its capacity to compromise both yield and quality make it a particularly intractable challenge across cropping systems. With chemical control options constrained by environmental and regulatory concerns, and host resistance often incomplete or unstable, sustainable and integrated disease management solutions have gained renewed urgency. This Research Topic brings together innovative studies aimed at improving the understanding of and control of *Fusarium* wilt through biological, ecological, molecular, and formulation-based approaches.

## Harnessing antagonistic fungi and soil amendments

A recurrent theme across multiple contributions is the potential of antagonistic fungi, particularly *Trichoderma* spp., to serve as biocontrol agents against *Fusarium* wilt. Pradhan et al. conducted a performance evaluation of novel tablet and powder formulations based on *T. viride* ITCC 7764 in chickpeas. These formulations demonstrated both superior biocontrol efficacy and agronomic benefits under field conditions compared to synthetic fungicides and talc-based formulations. These formulations were characterized by desirable physical properties and were enriched with volatile organic compounds including octan-3-one and 1-octen-3-ol, which are known for their antifungal activity. In addition, Long et al. isolated a promising *T. parareesei* strain (N4-3) from banana rhizospheres exhibiting potent antifungal activity against *F. oxysporum* f. sp. *cubense* TR4. This strain exhibited hyperparasitic behavior by deploying a range of cell wall-degrading enzymes, including 21 chitinases and 26 β-1,3-glucanases, resulting in severe structural disruption of the pathogen. Its application in pot trials significantly reduced the pathogen load and disease index while improving plant growth, highlighting its potential as an eco-friendly solution for controlling banana Fusarium wilt. Chen et al. identified two efficient biocontrol fungi *T. harzianum* and *Pestalotiopsis* sp. that can be used for *F. oxysporum* control in *Morinda officinalis*. To explore the control mechanisms of these biocontrol fungi, the authors used transcriptome sequencing analyses. They showed that expression levels in *M. officinalis* roots varied significantly following treatment with different biocontrol fungi, regulating signal transduction pathways and phytohormones related to pathogen resistance, such as the MAPK signaling pathway and the ethylene signaling pathway. This could improve the disease resistance and growth of *M. officinalis*. The biocontrol effects of some biological strains in field experiments are not ideal. Therefore, more efficient and locally adapted antagonistic strains still need to be selected. Zhou et al. identified the strain YNF2217 as the native endophytic fungus *Pochonia chlamydosporia.* This strain was selected from the root nodules of *Dolichos lablab* in the mulch of banana fields. This fungus demonstrated great potential for the management of *F. oxysporum* f.sp *cubense* race TR4 in banana. 

## Bioactive plant compounds and formulation innovations

The use of plant-derived compounds as green fungicides received significant attention. Saghrouchni et al. evaluated carvacrol, a phenolic monoterpenoid, for its dual function as a plant growth promoter and bio-fungicide in perennial ryegrass. In both *in vitro* and greenhouse experiments, carvacrol significantly suppressed disease symptoms caused by *F. oxysporum*, *F. solani*, and *F. nivale*, while simultaneously enhancing seedling vigor and root development. The compound’s preventive role and biostimulatory effects make it a promising tool for sustainable disease management. Mirmajlessi et al. proposed an implementation framework for evaluating the biocidal potential of essential oils against *F. oxysporum* in spinach. Moving from *in vitro* assays to *in planta* trials, the authors assessed key disease and growth parameters, providing strong evidence for essential oils as viable components in integrated pest management strategies. Together, these studies reflect a shift toward natural, multifunctional compounds capable of addressing plant health and productivity holistically.

## Exploring microbial interactions and community dynamics

Beyond direct biocontrol, insights into microbial ecology are vital for the sustainable management of *Fusarium* diseases. The work of Todorovic et al. examined the microbial consortia in suppressive soils using high-throughput sequencing and co-occurrence network analysis to identify dominant taxa associated with disease suppression in watermelon. Notably, *Trichoderma*, *Bacillus*, and *Pseudomonas* spp. emerged as key microbial players, suggesting that understanding soil microbial assembly and community structure can inform more targeted biocontrol interventions. In a parallel study, Choi et al. investigated *Fusarium graminearum* and *F. asiaticum* using community ecology frameworks. These frameworks revealed how stress tolerance and infectivity traits influence fungal community assembly. Phenotypic clustering, as evidenced by the Net Relatedness Index and the Nearest Taxon Index, was observed under stress and competition, indicating that local environmental pressures drive convergence among pathogenic strains. These findings underscore the importance of ecological filtering and resource competition in shaping pathogen populations, a critical consideration when designing long-term, evolutionarily robust control measures.

## Integrating host resistance and genomic approaches

While biocontrol is a key pillar of sustainable disease management, its success is often enhanced by host genetic resistance. In a genome-wide association study (GWAS) of 249 Canadian spring wheat lines, Semagn et al. identified multiple quantitative trait loci (QTLs) linked to Fusarium head blight resistance. Several of these QTLs overlapped with traits related to plant height and flowering time, reflecting the complexity and pleiotropy inherent in disease resistance breeding. Four QTLs were found to be consistently associated with disease incidence, severity, and visual rating index across environments, accounting for up to 33.2% of phenotypic variance. Conversely, Xu et al. were the first to create a high-density map of QTLs for yield-related traits, seed color and sesame Fusarium wilt disease using an F2 population. This study provides a solid basis for further genetic analyses of Fusarium wilt disease-related traits in sesame, including map-based gene cloning and marker-assisted selection breeding.

Host-induced gene silencing (HIGS) is a relatively new approach to managing plant diseases. Based on the pharmacological inhibition of calcineurin in *F. fujikuroi*, Hou et al. discovered that the calcineurin inhibitors FK506 and cyclosporin A can prevent the growth of *F. fujikuroi*. They then used an HIGS strategy to silence the calcineurin genes of *F. fujikuroi* in rice, finding that the transgenic rice seedlings exhibited enhanced resistance to infection by *F. fujikuroi*, which suggests that calcineurin is essential for F. fujikuroi to infect rice plants. Furthermore, these results provide valuable insights into the HIGS mechanism in plants and offer a promising approach to enhancing resistance against rice bakanae disease.

Comparative genomic and transcriptomic analyses of the different *F. oxysporum* races would reveal the most significant genetic differences between them, providing a better understanding of the pathogen’s evolution and potentially enabling more lasting resistance in crops. For this reason, Bates et al. presented the first full genome and transcriptome data of *F. oxysporum* f.sp. *lactucae* (Fola). This has enabled the identification of key differences in the effector repertoire and its expression in the globally significant Fola1 and Fola4 races in lettuce. Mestdagh et al. employed molecular detection methods (such as race-specific real-time PCR assays) to detect *F.* Fola races 1 and 4 in plant tissue and optimized sample preparation. These methods can now be used to study the pathogen’s spread and evaluate the impact of control measures, including various soil disinfestation methods, new resistant varieties, and alternative crops. This study provides the first quantitative data on the presence of Fola in greenhouse soil. Bhutia et al. employed an RNA sequencing-based approach to analyze the shoot transcriptome in order to understand the molecular mechanisms conferring resistance to *Fusarium* wilt infection in chickpeas. The authors concluded that efficient energy metabolism, activation of environmental adaptation mechanisms, and DNA stability were key to resistance against *Fusarium* wilt infection in chickpea genotypes.

On the other hand, charting the *F. oxysporum* f.sp. *pangenome* (the characterization of the complete set of genes of a species or an infra-species taxa) blueprint and studying its diversity is an essential and imperative initial step toward uncovering the mechanisms that underlie plant-fungal interactions, and devising new disease control strategies. Logachev et al. conducted an analysis of the pangenome of 13 *F. oxysporum* f.sp. *lini* isolates that belong to 4 clonal lineages with varying virulence, shedding new light on its genomic diversity. Moreover, these studies strongly support the hypothesis that *Fusarium oxysporum* isolates utilize different virulence factors during the infection of crops, enabling them to evade plant defense mechanisms. All these studies enrich the -omics tools available to crop breeders and support the integration of molecular and agronomic strategies for combating Fusarium diseases.

## Closing remarks

The studies presented in this Research Topic reflect the richness and diversity of approaches currently being pursued to manage *Fusarium* wilt diseases in different crops and agroecosystems. From novel *Trichoderma* strains and essential oil formulations to genomic mapping and microbial ecology, the Research Topic exemplifies the convergence of traditional plant pathology with modern tools in genomics, formulation science, and ecological modeling tools. Importantly, several contributions moved beyond proof-of-concept, offering scalable solutions with practical relevance under field conditions. The integration of biocontrol agents with plant-based compounds and host resistance strategies appears especially promising, pointing toward multi-layered approaches capable of withstanding pathogen evolution and environmental variability. Future research should emphasize long-term field validation, compatibility with agricultural inputs, and farmer-centered implementation strategies. As we continue to navigate the global challenge of sustainable food production, these interdisciplinary efforts will be indispensable.

